# A novel approach for simultaneous tibiofibular synostosis takedown and peroneus longus ligamentoplasty for posttraumatic tibiofibular synostosis: a case report and review of the literature

**DOI:** 10.1186/s13256-020-02397-7

**Published:** 2020-07-05

**Authors:** Michael Margulis, Lior Ben-Zvi, Itzhak Brook, Benjamin Bernfeld

**Affiliations:** 1 Lady Davis Carmel Medical Center, Mikhal Street 7, 3436212 Haifa, Israel; 2 Georgetown University Medical Center, 4431 Albemarle Street NW, Washington, DC 20016 USA

**Keywords:** Syndesmosis, Synostosis, Ligamentoplasty, Peroneus longus

## Abstract

**Introduction:**

A singular procedure involving both a distal tibiofibular synostosis resection with syndesmosis repair by peroneus longus ligamentoplasty has not been reported in the English literature. We report a case of simultaneous distal tibiofibular synostosis resection and syndesmosis stabilization by peroneus longus ligamentoplasty for the treatment of symptomatic distal tibiofibular synostosis formation, following neglected syndesmosis injury.

**Case presentation:**

A 42-year-old Caucasian man presented with ankle pain and painful range of motion 20 months following ankle trauma. Distal tibiofibular synostosis was identified, and our patient was successfully treated by simultaneous synostosis takedown and peroneus longus ligamentoplasty for distal tibiofibular syndesmosis repair.

**Conclusions:**

Our experience illustrates that in cases of painful posttraumatic distal tibiofibular synostosis, simultaneous synostosis resection with peroneus longus ligamentoplasty may show good clinical results.

**Level of evidence:**

5

## Background

Damage to the distal tibiofibular syndesmosis is a common complication of an ankle injury. It may follow an ankle fracture or a high ankle sprain.

A syndesmosis ankle injury (high ankle sprain) is challenging to diagnose, has a substantial recovery period, and may disrupt the ankle joint functioning. Effective evaluation and treatment for this injury require understanding of the functional anatomy of this region, and the etiologic factors [1–4].

Therapeutic strategies for the treatment of chronic syndesmotic lesions are described in the literature [5]. In this case, Simultaneous syndesmosis takedown and peroneus longus Ligamentoplasty has been utilized to treat this condition resulting in pain reduction and preservation of ankle range of motion.

A review of the English language literature did not reveal a reported case where synostosis resection with syndesmosis repair by peroneus longus ligamentoplasty was used simultaneously for synostosis surgical repair with successful results following ankle trauma.

We herewith report our unique experience in the treatment of a patient with symptomatic distal tibiofibular synostosis formation, using simultaneous distal tibiofibular synostosis resection and syndesmosis stabilization by peroneus longus ligamentoplasty.

## Case presentation

A 42-year-old Caucasian man, presented at our outpatient clinic in February 2015, complaining of left ankle pain that had been limiting his physical activity and affecting his gait for 20 months following ankle trauma (a “sprain”). His injury was treated with a walking cast for 6 weeks. The pain occurred during walking and was accompanied by ankle impingement and locking, and was aggravated during mild to moderate physical activity. There was no significant resting pain.

He has been a storage keeper at the local harbor for many years, had no prior medical problems, and did not smoke or use any medications.

A physical examination of his left foot and ankle showed no cutaneous pathology. There was tenderness around the distal tibiofibular syndesmosis and around the medial malleolus. There was full, yet painful passive ankle range of motion. The other results of his physical and neurological examination were normal.

**Fig. 1:**
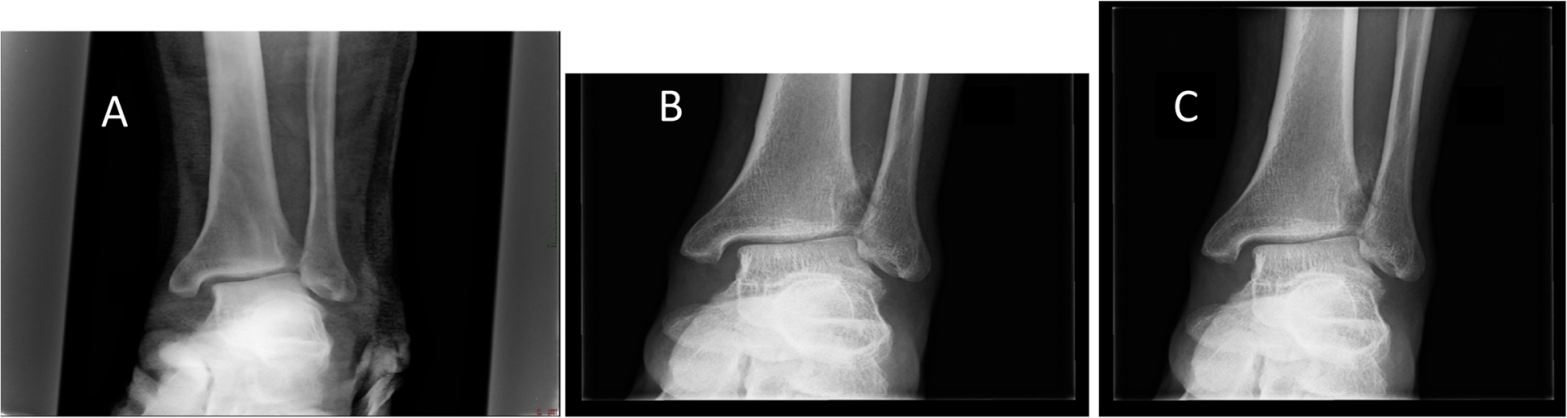
An antero-posterior radiographic view of our patient’s left ankle immediately (**a**), 6 (**b**), and 10 (**c**) weeks following his injury. Medial and superior tibio-talar space widening is evident on radiograph (**a**). Note the gradually appearing radio-opaque syndesmotic appearance (radiograph **b** and **c**)

**Fig. 2:**
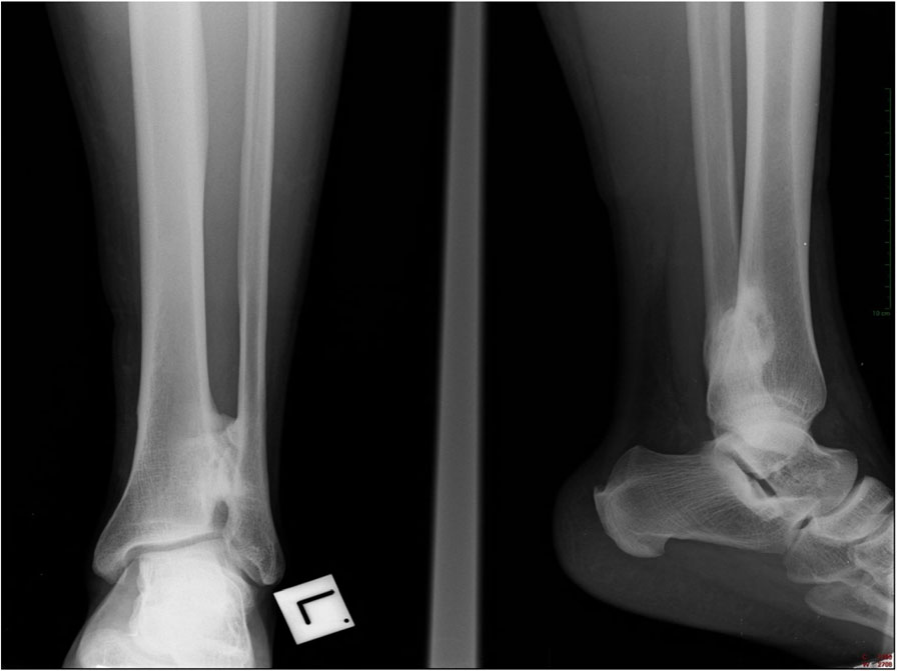
An antero-posterior and lateral radiographic view of our patient’s left ankle, taken 20 weeks after injury. Radio-opaque appearance is evident on the distal tibiofibular area, suggestive of tibiofibular synostosis formation

Left ankle radiography taken immediately after his injury suggested disruption of the distal tibiofibular syndesmosis, with medial clear space and tibiofibular widening. Later radiographies revealed gradual ossification with synostosis formation (Fig. 1). Knee radiographies did not show any knee injury or fracture of the proximal fibula (Fig. 2).

**Fig. 3:**
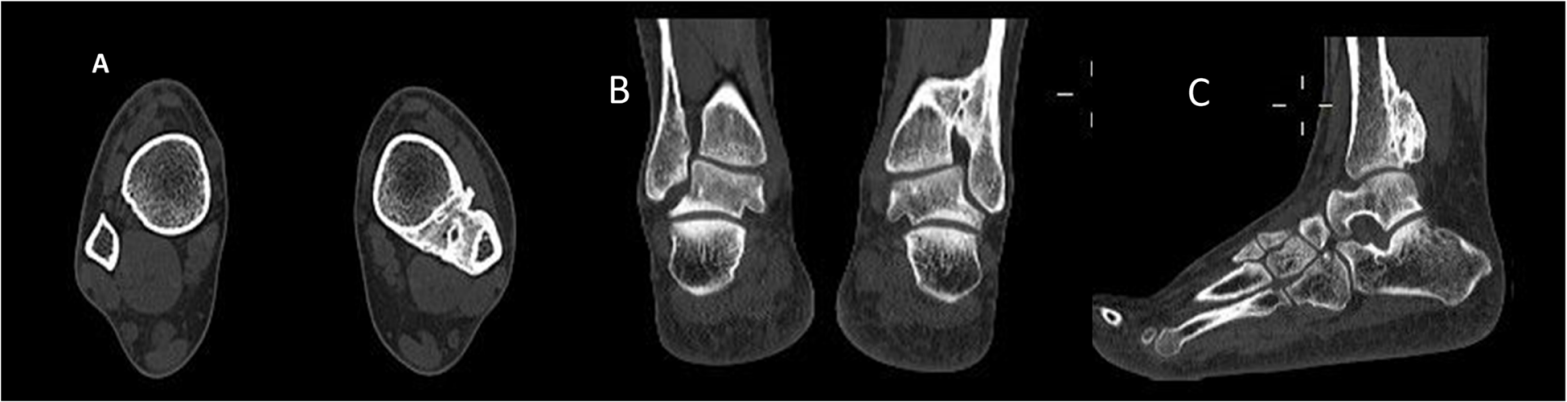
**a** to **c** 20 weeks following injury. Axial (**a**), coronal (**b**), and sagittal (**c**) computed tomography images of the patient’s ankle joint. Synostosis formation is evident

A computed tomography scan of the ankle, performed in our clinic, showed an ossified tibiofibular synostosis with a widening of the tibiofibular space and mild lateral translation of the talus (Fig. 3).

Surgical intervention was performed (by the first author) to treat the painful ankle joint and radiologic synostosis. The surgery included simultaneous resection of the osseous synostosis and syndesmosis repair by peroneus longus ligamentoplasty.

**Fig. 4:**
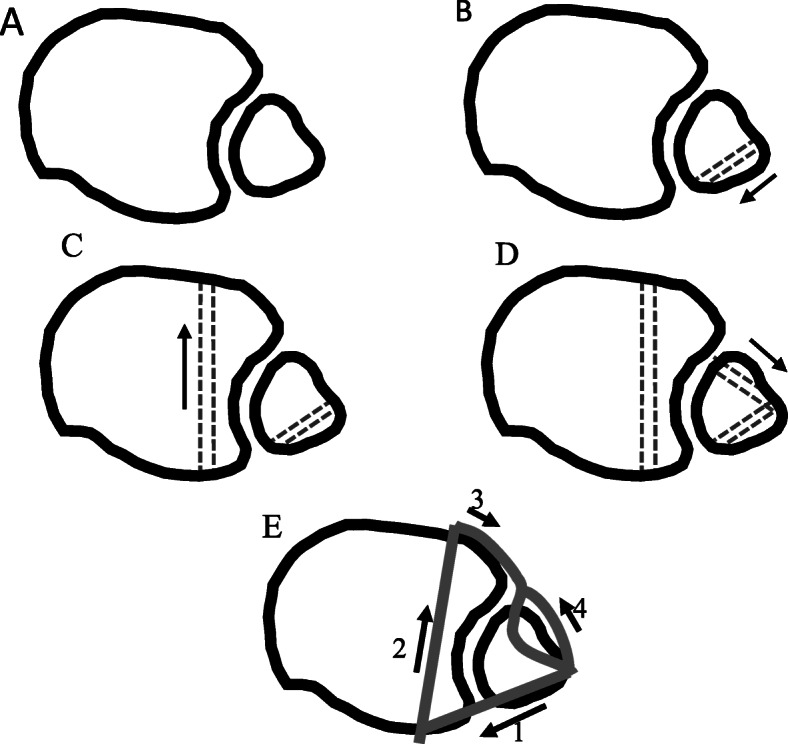
Surgical procedure. **a** Tibiofibular appearance as assessed with axial computed tomography. **b** First canal (3.5 mm in diameter) drilled into the distal fibula in posteromedial direction and 1.5 cm above the tibial plafond. **c** Second canal (3.5 mm in diameter) drilled through the tibia at the level adjacent to the fibular canal and directed anteriorly toward the anterior tibial cortex 1.5 cm above the syndesmosis. **d** Third canal (3.5 mm in diameter) drilled slightly cranially to the aforementioned fibular canal, and directed posterolaterally from the medial aspect of the fibula. **e** Surgical procedure illustrating the direction of the split peroneus longus tendon graft and numbered (1-4) according to the steps of its trajectory (4). Norkus and Floyd illustrates tendon free end anchored with a vicril suture

**Fig. 5:**
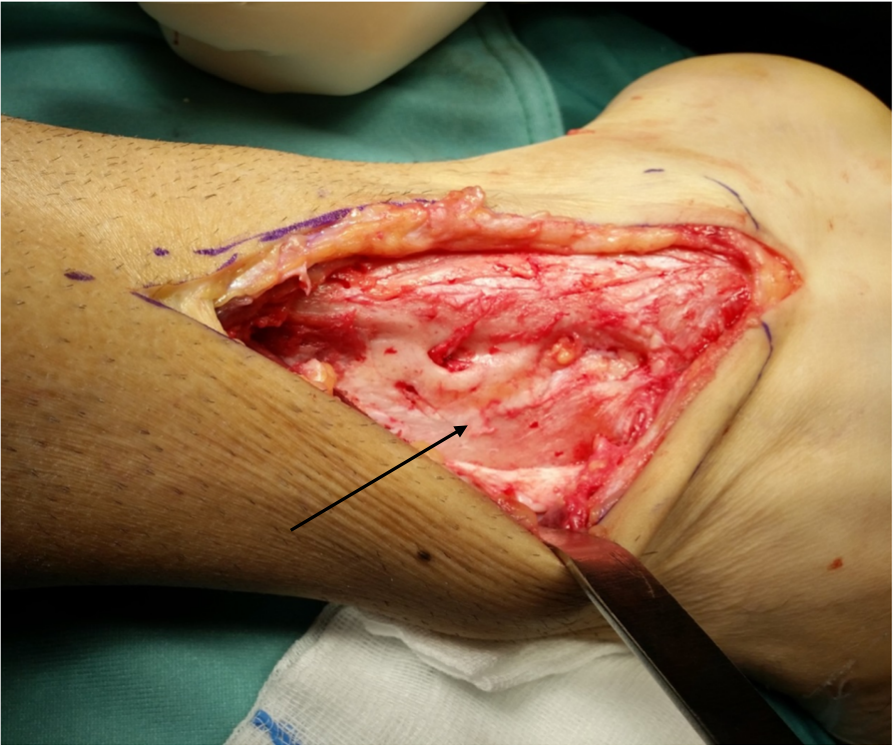
Intra-operative anterior view of osseous synostosis (marked with an *arrow*) of the tibiofibular syndesmosis

The surgery included the resection of the osseous synostosis, followed by a peroneus longus ligamentoplasty. The ligamentoplasty technique was based on the description by Rene Grass *et al.* [5] with a slight modification (Fig. 4). The operation was performed under general anesthesia and peripheral nerve ankle block. After applying a tourniquet below the knee, surgical curvilinear incision was performed through a lateral approach directly over the distal fibula. The osseous synostosis was exposed anteriorly (Fig. 5) and posteriorly and was completely excised. The distal tibia fibular groove was restored.

Bone wax was applied on the site of the resected synostosis. Under fluoroscopy imaging, the new free syndesmosis was temporarily reduced by a bone clamp.

**Fig. 6:**
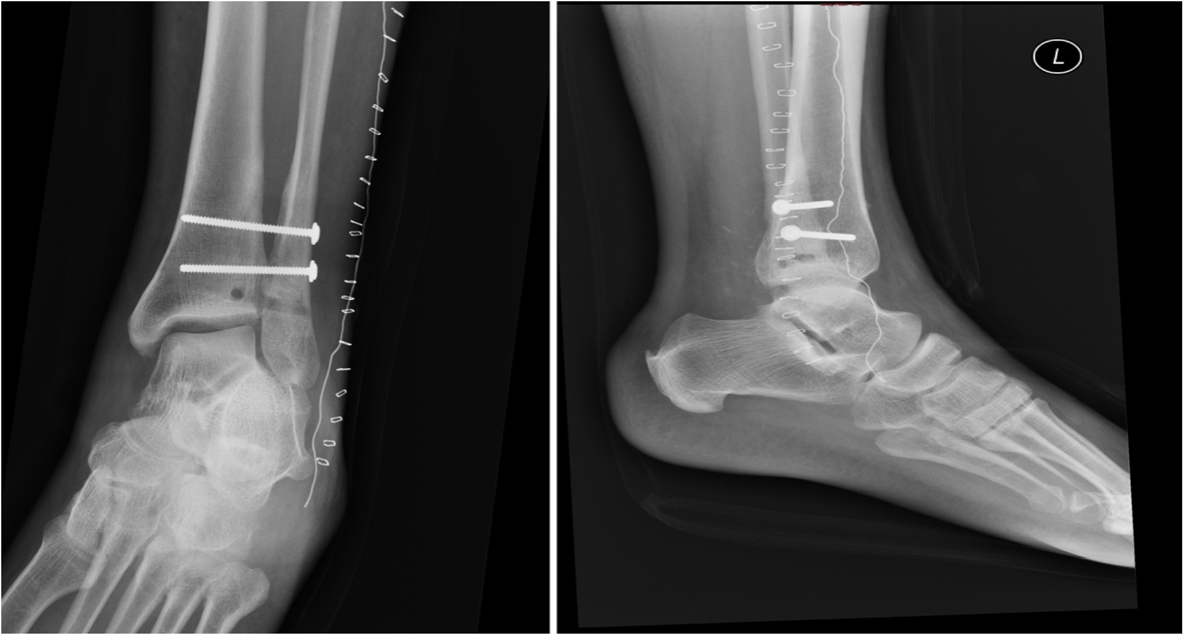
Postoperative radiograph at day 1. An internal rotation (mortise) and lateral view radiographs of the left ankle showing syndesmotic position and fixation. The tibiofibular osseous canals are also visible inferior to the bottom screw

**Fig. 7:**
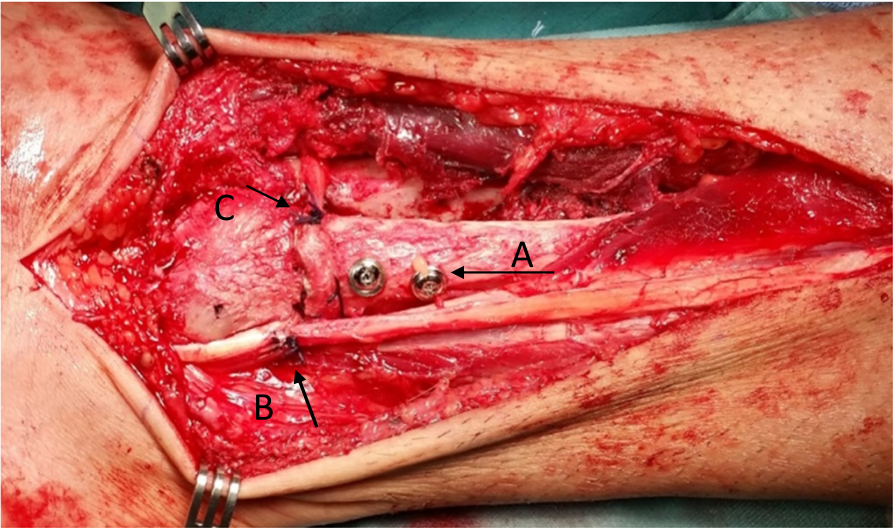
Intra-operative lateral view of the ankle. Visible are the syndesmotic screws ([**a**] *arrow*); vicril suture of split peroneus longus tendon graft at tendon divergence point ([**b**] *arrow*); vicril suture of the free tendon end anchored on the anterior aspect of the syndesmosis ([**c**] *arrow*)

The peroneus longus tendon was split into equal parts on a proximally to distally direction up to the level of distal tibiofibular syndesmosis. Augmentation with a 2 mm vicril suture was positioned at the site of the tendon splitting between the free tendon flap and its origin. A 4.5 mm canal was drilled into the distal fibula from a lateral to posterior direction and parallel to its horizontal axis 1.5 cm above the tibial plafond (Fig. 4). A second 4.5 mm canal was drilled through the tibia at the same level and directed from posterior, parallel to the tibial plafond, toward the anterior tibial cortex. A third 4.5 mm canal was drilled through the anteromedial aspect of the fibula directed to the lateral fibular cortex, slightly cranial to the level of the first canal. The free end of the detached proximal part of the peroneus longus tendon was secured by a Krakow suture and was passed through the posteriorly directed fibular canal (first canal). it was then introduced into the posterior opening of the tibial canal (second canal) and pulled anteriorly toward the anterior surface of the tibial cortex. The free end of the tendon was pulled out through another fibular canal using a wire passer directed posterolaterally (Fig. 4). Using a compressive clamp placed on the fibula and the tibia, the fibula was reduced into its tibial notch. The tendon was then tightened, and the syndesmosis stability was assured with an intraoperative external rotation (Frick) test. Two tricortical transfixation screws were positioned to serve as syndesmosis screws (Fig. 6). The tightened free end of the tendon was secured with a 2 mm Vicril suture on the anterior aspect of the syndesmosis to itself (Fig. 7).

Debridement and hemostasis were performed. A dose of 500 mg Aminocaproic acid was injected locally into the surrounding soft tissues. Intraoperative fluoroscopy showed a well-positioned, reduced syndesmosis (as shown in Fig. 6). After tourniquet deflation, meticulous hemostasis was performed. The wound was closed with subcutaneous Vicril sutures and skin staples. A sterile dressing and posterior cast splint were applied.

Our patient was followed up very closely with frequent visits to the clinic. The cast splint was changed to a plastic walking boot class one after 2 weeks. Active and passive range of motion were initiated 6 weeks after the surgery. Our patient was instructed not to bear any weight for 6 weeks and use the walking boot for weight-bearing for an additional 2 weeks. Full weight-bearing using an elastic ankle support was started 8 weeks postoperatively.

The syndesmotic screws were removed 15 weeks after the primary surgery.

A 13-month postoperative follow-up demonstrated our patient had a significant pain decrease with normal walking and a stable ankle. The sensation of impingement and locking has disappeared. There was no skin pathology, swelling or neuromuscular deficits. He could perform a single heel elevation on the operated side. Our patient was pleased with the results of the treatment and reported considerable improvement in his ankle function and pain, although he still had some pain during strenuous activity.

**Fig. 8:**
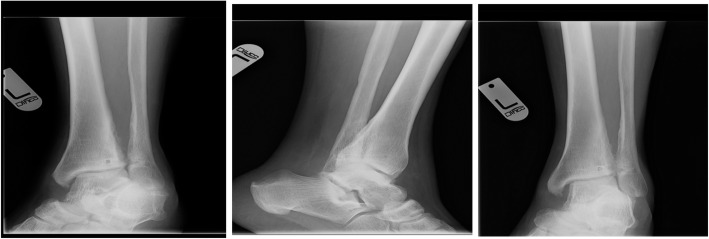
Postoperative radiographs at 13 months. An internal rotation (mortise), lateral and anterior-posterior view views of the left ankle (*left to right* respectively). The radiographs seem to exhibit normal syndesmosis appearance and position

The ankle’s range of motion is summarized in Table 1. Ankle radiographs taken 13 months postoperatively showed no signs of ossification or synostosis recurrence. An acceptable ankle joint position was evident, along with some degenerative changes and slight widening of the medial clear space (Fig. 8).

**Table 1:** Ankle range of motion in degrees according to postoperative week

Postoperative week	Degrees of dorsi flexion	Degrees of plantar flexion	Degrees of inversion	Degrees of eversion
12	5	30	10	5
30	15	45	10	10
56	20	50	10	20
Contralateral ankle	20	60	10	20

Our patient returned to work 7 months after his primary surgery. At that time, his American Orthopedic Foot and Ankle Society score (AOFAS) was 85 [6–11].

## Discussion

Our case report is the first description of a rare case, where synostosis resection with syndesmosis repair of the ankle, by peroneus longus ligamentoplasty was used simultaneously with successful results following trauma.

We have presented a detailed description of the surgical procedure of the simultaneous removal of the synostosis and peroneus longus ligamentoplasty for tibiofibular syndesmosis stabilization following ankle trauma.

The surgery resulted in obvious improvement in pain, along with the elimination of impingement and locking, to such an extent that our patient was able to perform at a similar level to his pre-injury state and feel pain-free. The range of motion of the injured leg became completely functional and was almost identical to the uninjured side (Table 1). As formation of synostosis following ankle injury is not uncommon, we suggest that synostosis resection with syndesmosis repair by peroneus longus ligamentoplasty should be kept in mind for the treatment of symptomatic patients.

There are several reports about the impact of tibiofibular synostosis of the ankle on the patient following conservative or operative treatment [5, 12–16]. This condition may cause pain, decreased ankle’s range of motion, and gait problems. Several of these reports noted that these injuries cause few symptoms in most patients and may not require any treatment. However, some reports supported a surgical approach for painful syndesmoses resulting in the alleviation of pain [14–16].

Several mechanisms, singular or combined, can cause syndesmosis injury; the most common one especially in combination with another mechanism, is an external rotation and hyper-dorsiflexion. These may cause a widening of the ankle mortise, which may result in disruption of the syndesmosis and the ankle instability [4].

Misdiagnosis or improper treatment of high ankle sprain might result in a serious disability that is difficult to treat.

Ankle syndesmosis features a complex mechanism of action. Ogilvie-Harris *et al.* [1, 2] studied the relative importance of each of the four syndesmotic ligaments. The anterior inferior tibiofibular, transverse and the interosseous ligaments were the three major ligamentous components which provide 90% of the overall stability against lateral fibular displacement. The percentage of contribution of each ligament was evaluated during 2 mm of lateral fibular displacement. The anterior inferior tibiofibular ligament provided 35%; the transverse (deep posterior) ligament, 33%; the interosseous ligament, 22%; and the superficial posterior inferior ligament, 9%. It was noted that Injury to one or more ligaments results in weakening, abnormal joint motion, and instability. Proper alignment and position of the syndesmosis are important for its intricate functioning. When the foot is moved from a plantar-flexed position to a dorsiflexed position, the joint permits approximately 1 to 2 mm of widening at the mortise. Dorsiflexion causes the fibula to externally rotate around in its tibial grove while plantar flexion causes the fibula to internally rotate. Rotation is about 3 to 5 degrees along a vertical axis in both cases [4]. The fibula migrates 2.4 mm inferiorly in weight bearing, and its motion deepens the ankle mortise and tightens the interosseous membrane. The tightness of the interosseous membrane also provides lateral support to the ankle [4].

When syndesmotic reconstruction is not possible or is contraindicated due to patient-related factors, tibiofibular arthrodesis is suggested. However, this procedure could produce a restriction of dorsiflexion [4]. Syndesmosis reconstruction was described utilizing Dacron tape, autologous peroneus brevis tendon, or free tendon graft (utilizing the long fourth toe extensor to restore the anterior and posterior tibiofibular ligaments) [5]. Also, split peroneus longus tendon was described by Grass *et al.* [5] to restore the anatomic fixation of the fibula to the tibia. Peroneus longus tendon, used as a split graft, may parallel the normal anatomic position of the syndesmosis, functionally reconstructing the anterior, posterior, and interosseous tibiofibular ligaments. Biomechanical testing of this technique is yet to be studied thoroughly. However, our case had a positive result as our patient was relieved of pain and had regained normal range of motion and gait. The clinical results are supported by radiographic evidence of regaining syndesmosis position.

## Conclusion

Posttraumatic synostosis formation with widening of the distal tibiofibular articulation may cause pain and hinder ankle function. Surgical treatment may be considered as the therapeutic option. Our case report suggests that simultaneous synostosis resection of the ankle with peroneus longus ligamentoplasty for distal tibiofibular syndesmosis stabilization show favorable results in reducing pain and improving impingement and locking sensations, while preserving the ankle’s range of movement.

## Data Availability

The data is available for review.
